# Surgical Outcomes Comparison of Spontaneous Middle Cranial Fossa Cerebrospinal Fluid Leaks: Systematic Review and Meta‐analysis

**DOI:** 10.1002/ohn.1279

**Published:** 2025-04-29

**Authors:** Dimitrios Spinos, Panagiotis Varoutis, Georgios Geropoulos, Georgios Vavoulis, Georgios Georgountzos, Nina Rafailia Karela, Manthia Papageorgakopoulou, Kyriacos Evangelou, Jameel Muzaffar, Wai Sum Cho

**Affiliations:** ^1^ Department of Cancer and Genomics School of Medicine, University of Birmingham Birmingham UK; ^2^ Department of Otolaryngology University Hospitals Birmingham NHS Foundation Trust Birmingham UK; ^3^ Department of Neurosurgery General Hospital of Thessaloniki Ippokratio, Thessaloniki Thessaloniki Greece; ^4^ West Hertfordshire Teaching Hospitals Watford UK; ^5^ Department of Neurosurgery General Attica Hospital “KAT” Athens Greece; ^6^ Department of Neurosurgery General Hospital of Nikaia “Agios Panteleimon” Athens Greece; ^7^ Department of Neurosurgery Carl Gustav Carus University Hospital, Technische Universität Dresden Dresden Germany; ^8^ School of Medicine University of Patras, Patras Patras Greece; ^9^ School of Medicine National and Kapodistrian University of Athens Athens Greece; ^10^ Department of Otolaryngology Nottingham University Hospitals NHS Trust Nottingham UK

**Keywords:** cerebrospinal fluid, meta‐analysis, middle cranial fossa, skull base, temporal bone

## Abstract

**Objective:**

Spontaneous cerebrospinal fluid (sCSF) leaks of lateral skull base have little consensus on optimal management. We synthesized and evaluated current literature via systematic and meta‐analysis to compare the success rates and complications of the different surgical techniques for middle cranial fossa (MCF) sCSF leak repair.

**Data Sources:**

MEDLINE, EMBASE, and Cochrane Library.

**Review Methods:**

Studies selected concerned surgical treatment of MCF sCSF leak. Data extracted included the following: study characteristics, patient characteristics, primary outcomes, and secondary outcomes.

**Results:**

From 297 repairs with a MCF approach, the complication rate was 16.2% (95% CI: 12.3%‐21.1%, *I* ^2^ = 0%, *P* = .052), compared to transmastoid (TM) 12.2% (95% CI: 6.7%‐ 21.2%, *I* ^2^ = 0%) in 82 repairs and for combined approaches 11.9% (95% CI: 4.2%‐29.6%, *I* ^2^ = 58%) in 98 repairs. The rate of recurrence with the MCF approach was 3.2% (95% CI: 1%‐6.4%, *I* ^2^ = 10%, *P* = .21) in 297 repairs, in the TM group the rate was 8.6% (95% CI: 4.7%‐15%, *I* ^2^ = 0%) in 125 procedures and 1.1% in the combined approaches group (0%‐4.5%, *I* ^2^ = 0%) in 139 procedures. Analysis of reoperation rates revealed a proportion of 0.9% (95% CI: 0%‐4.4%, *I* ^2^ = 51%) in 287 repairs via the MCF approach. Reoperation rate was 8.6% (95% CI: 4.7%‐ 15%, *I* ^2^ = 0%) in 125 repairs via TM and 1.1% (95% CI: 0%‐4.5%, *I* ^2^ = 0%) in 139 combined approach repairs.

**Conclusion:**

There is no statistically significant difference in the outcomes of repair techniques. Decision making for the preferred approach will be dependent on the location, size and number of the defects, hearing status, and in consultation with the patient.

Spontaneous cerebrospinal fluid (CSF) leaks, characterized by the unexpected escape of CSF through a defect in the dura mater, represent a significant clinical challenge.^1^ These leaks can lead to a range of symptoms, from mild headaches to severe neurological complications, significantly impacting patients' quality of life.^2^, ^3^ Understanding the varied approaches to manage and treat spontaneous CSF leaks is crucial, as the efficacy, risks, and recovery profiles differ markedly between methods.

Traditionally, conservative management, including bed rest, hydration, and caffeine intake, has been the first‐line approach.^4^ Although noninvasive, this approach is poorly evidence‐based and often yields variable results and may not provide long‐term relief. In cases where conservative treatment fails, more aggressive interventions are warranted. Medical management, such as administration of acetazolamide, has been used in selective patients as a single line of treatment, although in the majority of patients as an adjunct to surgical treatment.^5^

Surgical repair, although often avoided or delayed, offers a definitive solution by directly addressing the site of the leak.^6^ Advances in minimally invasive surgical techniques have improved outcomes, reducing recovery time and minimizing risks.^7^ Moreover, imaging technologies such as magnetic resonance imaging (MRI), computed tomography (CT), and potentially myelography play a pivotal role in accurately localizing the leak location, thereby enhancing the precision and success of surgical interventions.^8^, ^9^

The choice of treatment approach is influenced by several factors, including the severity and location of the leak, patient health status, and previous treatment responses. Comparative studies and clinical trials continue to refine these strategies, aiming to optimize patient outcomes and minimize complications.^10^, ^11^ Although previous studies in the literature have investigated the surgical outcomes of CSF leaks repair^6^, ^12^ and the repair outcomes of different materials for lateral spontaneous CSF leaks,^13^ no study has reviewed the outcomes of different surgical approaches for spontaneous CSF leaks of the lateral skull base.

The aim of this study is to conduct a systematic review of existing literature with meta‐analysis to compare the success rates and complications of alternative surgical techniques for spontaneous middle cranial fossa (MCF) CSF leak repair.

## Materials and Methods

### Study Design

This study was performed according to the Preferred Reporting Items for Systematic Reviews and Meta‐analyses (PRISMA) guidelines for systematic reviews.^14^ The protocol was registered in PROSPERO (International Prospective Register of Systematic Reviews) on June 21, 2022 (reference code: CRD42022340068).

Selection criteria were structured according to the PICO framework as follows:

− P (participants): Patients of any age and sex with spontaneous CSF (sCSF) leak of MCF.
− I (intervention): Surgical repair of sCSF leak.
− C (comparison): sCSF leak treated with extradural, intradural, or a combination of repairs.
− C (comparison): sCSF leak treated with an MCF, transmastoid (TM), or combination approach for repair.
− (Outcomes): Primary outcomes were cure/recurrence rates and complications. Secondary outcomes were length of stay (LOS) and revision rates.

Original clinical studies, published in English, investigating the spontaneous CSF leak of MCF were included. Exclusion criteria were the following:

i. Articles reporting nonspontaneous/acquired CSF leaks.
ii. Articles published in a language other than English.
iii. Narrative or systematic reviews and meta‐analyses.
iv. Animal and in vitro studies.
v. Case reports, errata, comments, perspectives, letters to the editor, and editorials that did not provide any primary patient data.
vi. Published abstracts with no available full text.
vii. Case series with less than five patients.
viii. Irrelevant studies (irrelevant population: anterior or posterior skull base CSF leaks, patients with previous history of trauma, neoplasm, chronic otitis media, and brain or skull base surgery).

### Search Strategy

Databases searched: MEDLINE via PubMed, EMBASE via Ovid, and Cochrane Library via Central databases were searched during the selection process (end‐of‐search date: April 2024).

The search strategy was developed with the guidance of a Clinical Librarian and included the following search terms:

(((((csf) OR ((cerebrospinal) AND (fluid))) AND (leak)) AND (spontaneous)) OR (meningocele) OR (encephalocele) OR (meningoencephalocele) OR ((spontaneous) AND (otorrh?ea))) AND (((lateral) AND (skull) AND (base)) OR ((temporal) AND (bone)) OR ((middle) AND (cranial) AND (fossa)))).

Snowball sampling was performed on the references of the included studies. Detailed search strategy for Ovid can be found in Supplemental Figure S1, available online. Two reviewers independently screened abstracts and subsequently full texts using the online COVIDENCE platform (Covidence systematic review software, Veritas Health Innovation, Melbourne, Australia; available at www.covidence.org). Disagreements were resolved by discussion, and if required arbitration by a third reviewer.

### Data Extraction

Two reviewers independently extracted relevant data through a standardized data extraction template. Any conflicts were resolved through discussion and consultation with a third reviewer. Data extracted included the following: (i) study characteristics (first author, year of publication, study design, and number of patients), (ii) patient characteristics (age, body mass index [BMI], sex, follow‐up, revision surgery, readmission, and type of surgical repair/approach), (iii) primary outcomes (recurrence, complications), and (iv) secondary outcomes (LOS, revision surgery).

### Quality of Evidence Assessment

Quality of included studies was assessed employing the National Heart, Lung and Blood Institute (NHLB) Assessment Tool for case‐control studies (12 criteria), observational cohort and cross‐sectional studies (14 criteria), and case series studies (9 criteria). Studies were classified as poor, fair, or good quality for each item of the assessment tool. There is no specific score threshold that would categorize a study as poor, fair, or good; thus, the assessment was subjective, and any discrepancies were resolved by a third reviewer. Before the quality assessment stage, it was decided that “poor” studies would be excluded.

### Statistical Analysis

Obtainable data from included studies regarding approach‐based outcomes were further investigated through a single‐arm proportional analysis. Statistical analysis was performed among studies reporting outcomes for one specific approach or providing individual patients' data that allowed data extraction for approach‐specific outcomes. Articles reporting pooled complications, recurrence, or revisions were not analyzed. Statistics were analyzed with R (version 4.4.2, R Foundation for Statistical Computing). A single‐arm proportional analysis with 95% confidence intervals was used. A random effects model with logit and double arcsine transformation was utilized when the main proportion was <0.2 or equal to zero, respectively. The inverse variance method was applied to account for individual study weights. When the number of analyzed studies was ≥10, publication bias was assessed through funnel plot and Egger's test, whereas heterogeneity among included studies was evaluated with *I* ^2^ and Baujat plots.

## Results

### Study Selection and Characteristics

**Figure 1:**
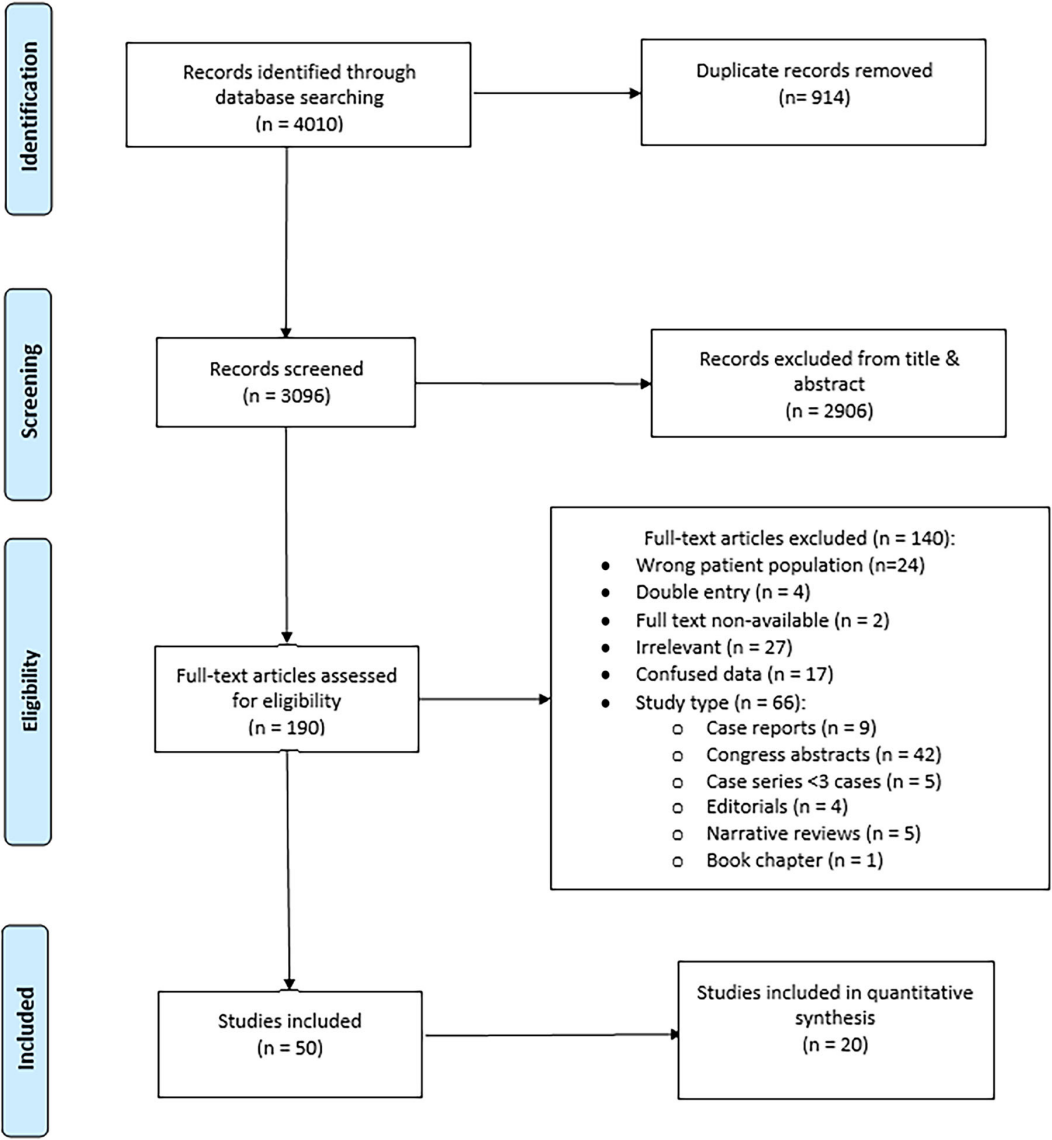
Preferred Reporting Items for Systematic Reviews and Meta‐analyses flowchart of included studies.

A systematic search identified 3096 abstracts. After the removal of duplicates and title/abstract review, 190 articles underwent full‐text review. Of these, 50 articles focusing on the surgical treatment of patients with spontaneous MCF CSF leak were included in this study. This process is described in more detail in the PRISMA flowsheet (Figure 1).

### Baseline Characteristics

Patient characteristics and demographics from included studies can be found in Table 1. From the 50 studies, 1475 patients or 1544 ears were retrieved, and 1529 repairs were reported. Mean age was 57.2 years, mean BMI was 36.2 kg/m^2^, and there was a slight gender predilection in favor of female patients (n = 823, 55.8%). The mean length of postoperative hospital stay was 3.8 days, the mean follow‐up was 24.7 months, and 150 complications were reported.

**Table 1:** Basic Characteristics of Included Studies

First author	Year of publication	Country	Study design	Patients (N)	Ears (N)	Female patients (N, %)	Age, y (mean, SD)	BMI, kg/m² (mean, SD)	Follow‐up, mo (mean, SD)	Recurrence (N, %)	Length of stay, d (mean, SD)
Ahmed^15^	2016	Canada	Case series	5	5	3 (60%)	53.6 (8.2)	30.8 (2.8)	5 (N/A)	0	N/A
Alwani^16^	2019	United States	Cohort study	24	27	18 (75%)	60.8 (9.2)	37.8 (6.8)	8.6 (10.3)	0	N/A
Alwani^17^	2021	United States	Cohort study	38	45	26 (68.4%)	54.7 (12.3)	38.5 (8.7)	13.5 (12.9)	2 (5.5%)	3.1 (1.6)
Brenet^18^	2019	France	Case series	41	44	24 (58.5%)	57.2 (19.3)	33.1 (8.6)	61 (N/A)	2 (4.5%)	3.6, 5.8, 4.8^a^ (N/A)
Brown^19^	2004	United States	Case series	9	9	3 (33.3%)	57.8 (8.4)	N/A	14.7 (12.7)	N/A	N/A
Carlson^20^	2013	United States	Case series	35	35	25 (71.4%)	57.4 (12.2)	37 (14.7)	N/A	4 (11.4%)	N/A
Cheng^21^	2019	United States	Case series	58	58	29 (50%)	58 (N/A)	32 (N/A)	N/A	2 (3.4%)	N/A
Cooper^22^	2020	United States	Case series	43	45	24 (55.8%)	57.4 (12.9)	35 (7.8)	N/A	N/A	N/A
Cumpston^23^	2023	United States	Cohort study	13	13	9 (69.2%)	55.5 (8.8)	45.4 (15.1)	N/A	0	N/A
Goddard^24^	2010	United States	Case series	23	23	13 (56.5%)	60.2 (9.4)	36.3 (6.7)	N/A	3 (13%)	N/A
Gonen^25^	2015	Israel	Case series	9	10	7 (77.8%)	56.9 (16.9)	33.4 (3.7)	19.5 (N/A)	0	N/A
Grinblat^26^	2018	Italy	Case series	61	72	25 (41%)	57.1 (N/A)	N/A	34.8 (N/A)	0	N/A
Gubbels^27^	2007	United States	Case series	15	16	10 (66.7%)	56.9 (18.7)	N/A	N/A	1 (6.7%)	N/A
Hendriks^28^	2022	Australia	Case series	38	38	N/A	N/A	N/A	N/A	2 (5.3%)	N/A
Hentati^29^	2023	United States	Case series	47	47	N/A	N/A	N/A	N/A	9 (19.1%)	N/A
Hoang^30^	2018	United States	Case series	21	22	18 (85.7%)	57 (9.9)	35.4 (9.5)	14.9 (21.4)	0	N/A
Hwa^31^	2022	United States	Case series	43	43	22 (51.2%)	58.6 (10.9)	35.4 (7.6)	48.6 (22)	3 (7%)	N/A
Khanna^32^	2022	United States	Case series	60	60	35 (58.3%)	58.4 (11.9)	35.8 (9.3)	73.3 (77.4)	1 (1.7%)	4.2 (N/A)
Kim^33^	2014	United States	Case series	16	16	9 (56.3%)	57.7 (11.3)	40.7 (8.7)	15.4 (15.5)	1 (6.3%)	N/A
Kutz^34^	2008	United States	Case series	17	19	12 (70.6%)	61 (N/A)	34.2 (N/A)	11 (N/A)	0	N/A
Kutz^35^	2018	United States	Case series	50	55	37 (74%)	57.2 (N/A)	35 (N/A)	20.4 (N/A)	5 (10%)	N/A
Leonetti^36^	2005	United States	Case series	48	51	28 (58.3%)	45.7 (N/A)	N/A	58.8 (N/A)	2 (3.9%)	N/A
Lundy^37^	1996	United States	Case series	11	11	4 (36.%)	57.1 (15.8)	N/A	N/A	N/A	N/A
Markou^38^	2011	France	Case series	12	14	6 (50%)	55.4 (12.3)	N/A	N/A	0	N/A
Mayeno^39^	2004	United States	Case series	5	6	2 (40%)	59 (11.2)	N/A	N/A	0	3.3 (2.8)
May^40^	1995	United States	Case series	12	12	5 (41.7%)	51.9 (19.6)	N/A	N/A	1 (8.3%)	N/A
Miller^41^	2023	United States	Case series	89	92	51 (57.3%)	59.6 (12.9)	34.9 (8.3)	16.3 (17.9)	4 (4.5%)	1.3 (1.5)
Mostafa^42^	1997	Egypt	Case series	14	14	0	54.2 (N/A)	N/A	14.4 (N/A)	2 (14.3%)	N/A
Nahas^43^	2008	United States	Case series	15	15	10 (66.7%)	58.5 (14.2)	N/A	N/A	N/A	N/A
Nelson^44^	2016	United States	Case series	60	65	41 (68.3%)	57.5 (11.4)	37.5 (8.7)	19.5 (N/A)	5 (7.7%)	4.8 (2)
O'Connell^45^	2017	United States	Case series	13	13	N/A	N/A	N/A	N/A	0	N/A
Oliaei^46^	2012	United States	Case series	15	18	12 (80%)	61.2 (12)	N/A	12.7 (17.8)	1 (5.6%)	N/A
Pappas^47^	1995	United States	Case series	12	12	8 (66.7%)	63.6 (12.4)	N/A	N/A	1 (8.3%)	N/A
Patel^48^	2000	United States	Case series	7	7	4 (57.1%)	55.4 (10.2)	N/A	N/A	0	N/A
Perez^49^	2017	United States	Case series	28	33	13 (46.4%)	52 (13)	35.4 (7.3)	33 (N/A)	8 (24.2%)	3 (3.9)
Quimby^50^	2023	United States	Case series	79	87	49 (62%)	57.5 (10.7)	N/A	17.4 (19.6)	N/A	N/A
Rao^51^	2005	United States	Case‐control study	10	11	9 (90%)	57.9 (14)	N/A	19 (N/A)	3 (25%)	N/A
Sanna^52^	2009	Italy	Case series	25	25	N/A	N/A	N/A	N/A	0	N/A
Schwartz^53^	2021	United States	Case series	10	10	N/A	57.3 (13.5)	46.2 (9.9)	22.2 (5.5)	0	N/A
Scullen^54^	2021	United States	Case series	5	8	5 (100%)	56.2 (4.9)	32.2 (6.7)	11.3 (4.7)	0	4.8 (0.4)
Shah^55^	2023	United States	Cohort study	55	60	30 (54.5%)	54.5 (9.0)	35.6 (8.5)	26.1 (27.2)	5 (8.3%)	N/A
Stevens^56^	2017	United States	Case series	28	31	19 (67.9%)	60 (9.3)	35.9 (10.1)	25.5 (N/A)	1 (3.2%)	N/A
Stevens^9^	2016	United States	Case series	48	48	39 (81.3%)	60.1 (1.8)	35.7 (1)	23.1 (N/A)	9 (18.8%)	N/A
Stucken^57^	2012	United States	Case‐control study	11	11	8 (72.7%)	61.6 (8.9)	33.4 (6)	32.8 (53.6)	1 (9.1%)	N/A
Swanson^58^	2022	United States	Case series	39	43	18 (46.2%)	56.3 (11.7)	35.8 (8.3)	N/A	6 (14%)	4.6 (10.7)
Symms^59^	2023	United States	Case series	52	54	36 (69.2%)	61.4 (12.1)	35 (8.3)	19.7 (N/A)	3 (55.6%)	N/A
Valtonen^60^	2001	Finland	Case series	5	5	3 (60%)	57.8 (10.9)	N/A	N/A	1 (20%)	N/A
Vivas^61^	2014	United States	Case series	32	34	22 (68.8%)	56 (N/A)	35 (8.5)	23 (N/A)	3 (8.1%)	N/A
Walia^62^	2021	United States	Case series	28	28	22 (78.6%)	54 (14.6)	40.1 (14.1)	N/A	1 (3.6%)	2 (0.5)
Yancey^63^	2020	United States	Case series	41	24	30 (73.2%)	56 (13)	36.9 (8)	N/A	1 (2.4%)	N/A

^a^ Reported individually per technique: transmastoid, middle cranial fossa, and combined, respectively.

Three principal approaches were identified:

1. MCF approach with craniotomy,^10^, ^13^, ^14^, ^15^, ^16^, ^17^, ^18^, ^19^, ^20^, ^21^, ^22^, ^23^, ^24^, ^25^, ^26^, ^27^, ^28^, ^29^, ^30^, ^33^, ^34^, ^35^, ^36^, ^37^, ^38^, ^39^, ^40^, ^44^, ^46^, ^47^, ^49^, ^50^, ^52^, ^53^, ^57^, ^58^, ^59^, ^64^
2. TM approach with mastoidectomy,^9^, ^18^, ^21^, ^22^, ^24^, ^26^, ^28^, ^29^, ^31^, ^33^, ^34^, ^35^, ^38^, ^39^, ^40^, ^42^, ^46^, ^47^, ^49^, ^50^, ^51^, ^52^, ^54^, ^55^, ^59^, ^60^, ^61^, ^63^, ^64^ and
3. Combination of MCF and TM.^10^, ^16^, ^17^, ^18^, ^19^, ^20^, ^22^, ^24^, ^26^, ^28^, ^29^, ^31^, ^32^, ^33^, ^34^, ^36^, ^37^, ^39^, ^43^, ^44^, ^45^, ^46^, ^47^, ^49^, ^50^, ^51^, ^52^, ^55^, ^57^, ^59^, ^60^, ^64^, ^65^

The most frequently used approach was MCF (48%, 735/1529 repairs), followed by TM (26%, 401/1529 repairs) and combined (24%, 358/1529 repairs), 35/1529 repairs (2%) were uncategorized and treated with MCF and combined without the authors disclosing which techniques were employed on which or how many patients.^20^ Two‐fifths of studies reported the use of multiple approaches. Bilateral lateral skull base sCSF leaks were relatively uncommon,^66^ with an incidence of just higher than 6% in our study. Although the subtle gender predilection (almost 6% higher incidence in females) is not a major finding in our review, it is observed that sCSF leak patients seemed to be relatively young (<60 years old)^67^ and obese.^68^ A mean recurrence rate of 6.9% was noted across all studies. Weighted mean LOS was 4.2 (SD 0.9) days for MCF patients,^17^, ^18^, ^39^, ^44^, ^49^, ^58^ 3.1 (SD 1.1) days for TM patients,^18^, ^39^, ^49^, ^54^ and 3.7 (SD 1.1) for combined approach patients.^18^, ^32^, ^49^, ^62^

### Approach‐Related Outcomes

**Figure 2:**
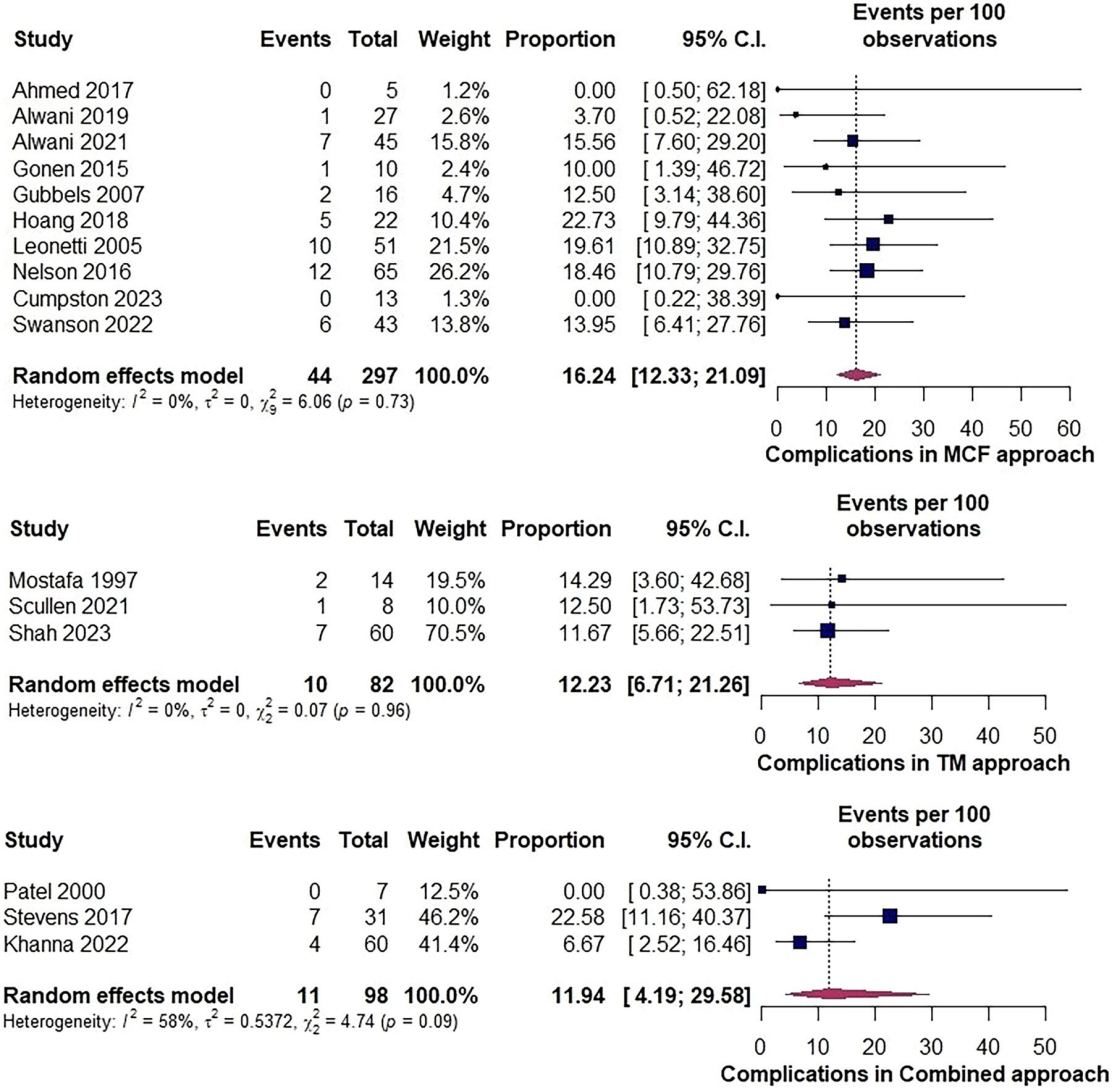
Forest plot demonstrating complication rates among approaches. MCF, middle cranial fossa; TM, transmastoid.

From 297 repairs with a MCF approach, the complication rate was 16.2% (95% CI: 12.3%‐21.1%, *I* ^2^ = 0%, with no asymmetry in visual assessment of funnel plot and Egger's regression test did not confirm publication bias, *P* = .052, Supplemental Figure S2, available online), compared to TM 12.2% (95% CI: 6.7%‐21.2%, *I* ^2^ = 0%) in 82 repairs and for combined approaches 11.9% (95% CI: 4.2%‐29.6%, *I* ^2^ = 58%) in 98 repairs (Figure 2). To investigate heterogeneity in the latter, sensitivity analysis with a random effects model was performed, excluding one study at a time (Supplemental Figure S3, available online). Rates varied between 7% and 21% with a rate of 7% (95% CI: 3%‐16%, *I* ^2^ = 0%) and 21% (95% CI: 10%‐37%, *I* ^2^ = 0%) when omitting Stevens et al^56^ or Khanna et al,^32^ respectively. Baujat plot also showed these studies as outliers (Supplemental Figures S4 and S5, available online). Visual assessment of the funnel plot (Supplemental Figure S6, available online) was not suggestive of asymmetry, supported by Eggers's regression test (*P* = .56).

As far as the nature of these complications is concerned, for the MCF approach, the following were reported: wound infections (n = 5, 1.2%), seizures (n = 5, 1.2%), facial weakness (n = 5, 1.2%), expressive aphasia (n = 4, 1%), seromas (n = 3, 0.7%), subdural hematoma (SDH) (n = 3, 0.7%), prolonged headaches (n = 2, 0.5%), eustachian tube dysfunction (n = 2, 0.5%), sensorineural hearing loss (n = 2, 0.5%), atrial fibrillation (n = 2, 0.5%), edema (n = 2, 0.5%), conductive hearing worsening (n = 1, 0.2%), ileus (n = 1, 0.2%), diabetic nephropathy (n = 1, 0.2%), metabolic encephalopathy (n = 1, 0.2%), corneal abrasion (n = 1, 0.2%), brain abscess (n = 1, 0.2%), meningitis (n = 1, 0.2%), wound dehiscence (n = 1, 0.2%), attic retraction pocket, CSF collection (n = 1, 0.2%), tension pneumocephalus (n = 1, 0.2%), and subtemporal extradural hematoma (n = 1, 0.2%).

The TM approach was correlated with hearing loss (n = 4, 3.2%), blurry vision (n = 3, 2.4%), headaches (n = 2, 1.6%), wound hematoma (n = 1, 0.8%), post‐op infection (n = 1, 0.8%), and empyema (n = 1, 0.8%).

Finally, the combined approach was related to wound infection (n = 3, 2.3%), hearing loss (n = 1, 0.7%), ossicular (n = 1, 0.7%), anterior fossa leak (n = 1, 0.7%), wound CSF leak (n = 1, 0.7%), meningitis (n = 1, 0.7%), aphasia (n = 1, 0.7%), and seizure (n = 1, 0.7%).

### Recurrent Rates

**Figure 3:**
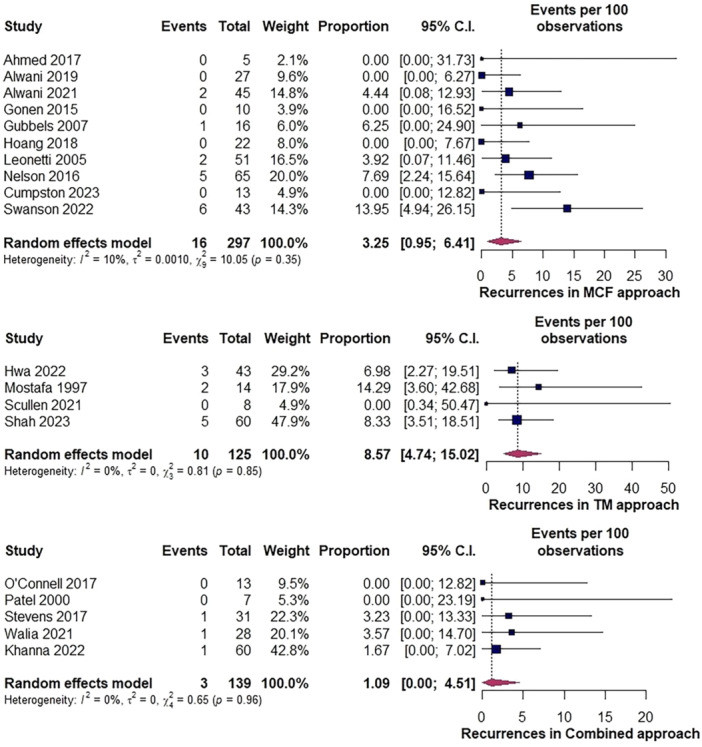
Forest plot depicting recurrence rates among approaches. MCF, middle cranial fossa; TM, transmastoid.

The rate of recurrence with the MCF approach was 3.2% (95% CI: 1%‐6.4%, *I* ^2^ = 10%). Funnel plot analysis revealed slight asymmetry with Egger's regression test negative for significance in asymmetry (*P* = .21, Supplemental Figure S7, available online) in 297 repairs. In the TM group, the rate was 8.6% (95% CI: 4.7%‐15%, *I* ^2^ = 0%) in 125 procedures and 1.1% (0%‐4.5%, *I* ^2^ = 0%) in 139 combined approaches (Figure 3).

Analysis of reoperation rates revealed a proportion of 0.9% (95% CI: 0%‐4.4%, *I* ^2^ = 51%) in 287 repairs via the MCF approach. Heterogeneity was investigated by sensitivity leave‐one‐out analysis (Supplemental Figure S8, available online) with rates varying from 0% to 1% with a rate of 0% (95% CI: 0%‐1%, *I* ^2^ = 0%) when omitting Swanson et al.^69^ Baujat plot identified Swanson et al as the main outlier study (Supplemental Figures S9 and S10, available online). Visual investigation of funnel plot revealed slight asymmetry (Supplemental Figure S11, available online); however, Egger's regression test did not confirm publication bias (*P* = .8).

**Figure 4:**
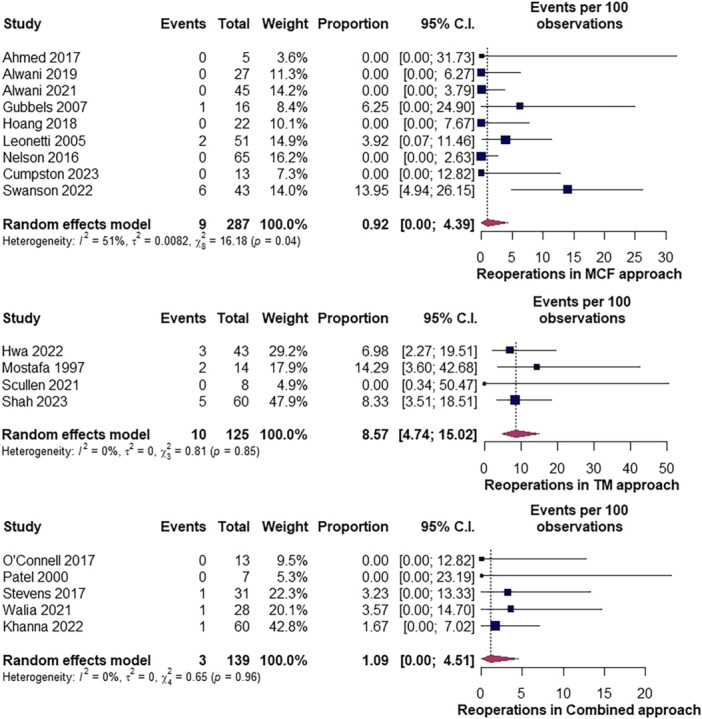
Forest plot illustrating reoperation rates among approaches. MCF, middle cranial fossa; TM, transmastoid.

Reoperation rate was 8.6% (95% CI: 4.7%‐15%, *I* ^2^ = 0%) in 125 repairs via TM and 1.1% (95% CI: 0%‐4.5%, *I* ^2^ = 0%) in 139 combined approach repairs (Figure 4).

The mean time of a CSF leak recurrence was 14.4 months (SD 12.9), and their management was variable across different institutions. Treatment options reported included conservative means, such as acetazolamide,^18^ lumbar drains,^44^ or shunts,^18^ in conjunction with or without revision surgery.

### Quality Assessment

The NLHB quality assessment tool was used to assess methodological quality, with studies rated as poor, fair, or good. Overall, 28% of the studies were rated as fair, and the majority (72%) were rated as good quality studies.

## Discussion

### Approach Outcomes

This systematic review and meta‐analysis shows high levels of success for all surgical approaches evaluated. No statistically significant differences were found to support the efficacy of any of the MCF, TM, or combined approaches for lateral sCSF leaks over the others, with respect to recurrence, reoperation, and complication rates. These results align with previous evidence in the literature^29^ supporting no significant difference between the TM and MCF sCSF leak recurrence rates. Although the overall benefit between these techniques did not reach statistical significance in our analysis, some individual studies have suggested benefits of one approach over the other. Variations in the complication and recurrence rates were noted across the different groups. Complication rates were the highest across the MCF group (16.2%), compared to the TM (12.2%) and the combined approaches groups (11.9%). On the other hand, recurrence rates were lower among the MCF group (3.2%) compared to the TM group (8.6%) and the lowest among the patients of the combined approaches group (1.1%). This was slightly different compared to the previous results of Gioacchini et al^12^ who also performed subgroup analysis for the different repair approaches. This increase in the recurrence rates of TM approaches may be driven by the underlying pathology, as we studied only spontaneous CSF leak cases, who are often associated with higher intracranial pressures^70^ or by a paradigm shift in the criteria driving the patient selection for a TM approach over an MCF or combination approaches.^49^ Across the literature, different authors reported their preference for the use of one of the approaches over the others, drawing conclusions from their own series and outcomes.

Alwani et al demonstrated that the MCF approach offered significantly improved audiological outcomes in their cohort,^16^ previously highlighted by Leonetti et al^36^ in their cohort reporting a 96.1% sCSF leak resolution rate. Gonen et al^25^ supported the use of MCF over the TM approach, highlighting high rates of complete resolution and a low risk of craniotomy‐related complications. Gubbels et al^27^ also recommended the MCF approach (with or without a concomitant TM approach) in the presence of multiple defects in the tegmen tympani. The MCF approach offers extensive visualization and access for instrumentation of the middle fossa floor, facilitating easier graft placement and skull base reconstruction.^58^ Although this approach is associated with potential complications such as postoperative CSF leak or SDH, most of these could be managed safely.^35^, ^44^

On the other hand, the TM approach offers faster postoperative recovery and reduced length of hospitalization; Perez et al^49^ presented one of the largest case series, reporting LOS of 1.6 days, versus 6.3 for the MCF approach. Both tegmen mastoideum and tympani were reported to be easily accessible through a TM approach, although the success rate of primary repair via TM compared to MCF was reported to be lower, due to the complexity of each of these patients' presentation.^49^ Shah et al^55^ presented the largest cohort to date demonstrating successful repair of lateral skull base sCSF leaks in 92% of cases, via a TM approach, when they combined it with the use of hydroxyapatite cement in the defect repair. Several authors suggested criteria that could help in the selection of one technique over the other for a particular patient. Mayeno et al^39^ recommended the use of a TM approach for tegmen mastoideum defects smaller than 1 cm. For more anteriorly located lesions or defects larger than 1 cm, they suggest an MCF approach or a combination of techniques. Oliaei et al^46^ later supported a TM approach for any posterior fossa or tegmen mastoideum defects as a first‐line approach, independent of their size or number, due to the relatively lower technical demand, limited morbidity, and shorter hospitalization, while recommending an MCF approach for tegmen tympani or petrous apex defects. Lastly, Pelosi et al^71^ opted for a TM approach for most of their patients, avoiding it only for multiple, large, or medially located defects, as well as revision surgery.

A combined TM and MCF approach is also utilized, and multiple authors have explored its effectiveness and safety. O'Connell et al^45^ reported no recurrence and minimal postoperative complications in a follow‐up period of 8 months, utilizing a combined technique with a composite intracranial extradural graft with a “pull‐through” suture. Stevens et al^56^ reported a 3.5% postoperative leak rate and good perioperative outcomes from the routine use of lumbar drain along with a combined approach. However, as we demonstrated, across the literature, these patients had a longer postoperative stay compared to the TM approach.

The recurrence rates in this study are similar to the ones previously reported in the literature.^6^ The use of lumbar drains to prevent recurrence, while widely practiced across several institutions, has limited evidence supporting it and remains controversial.^72^, ^73^ Among the articles of our review, Hentati et al reported lower recurrence rates amidst patients that had a lumbar drain placed postoperatively.^29^ On the other hand, Perez et al^49^ did not report any significant clinical decision driven by the intracranial pressure measurements, nor was the inpatient LOS reduced.

### Indications for Approach

Typical indications for sCSF repair are most commonly for infection risk mitigation (meningitis), which if untreated could reach an incidence up to a 20%,^16^, ^38^ management of chronic otorrhea, and treatment for associated hearing loss^16^ as highlighted in the works of Mostafa,^42^ Markou et al,^38^ and Alwani et al.^16^ This systematic literature review demonstrated a lack of high‐quality evidence currently available in the literature, lacking any large prospective studies or randomized trials, with most data derived from retrospective studies (49 out of 50), aligning with the current absence of standardized practice and guidelines in the management of lateral skull base sCSF leaks. Although factors such as surgeon's expertise, patient age, comorbidities, history of previous repairs, location and size of the defect, and preexisting hearing loss often influence the decision on preferred approach, this systematic review demonstrated no statistically significant differences in recurrence, complications, or reoperation rates to support the superiority of one approach over another.

The TM approach was typically recommended for posterolateral/posterior defects of the tegmen mastoideum,^49^ particularly if they are singular and small (<2 cm),^33^, ^59^, ^74^ whereas some described successful repairs of larger defects of the tegmen mastoideum.^59^, ^75^, ^76^ Moreover, the TM approach offered better visualization to the structures of the middle ear, when their integrity was to be protected, such as the ossicles for patients with preserved hearing or the tympanic segment of the facial nerve, when dehiscence of the canal is confirmed or suspected.^59^ However, a tegmen tympani defect would be challenging to manage in the presence of an intact ossicular chain. TM was from the preference the preferred approach of some authors when concurrent posterior fossa leak was suspected,^59^ offering technical simplicity and providing access to the posterior fossa while remaining extradural and minimizing temporal lobe retraction, as highlighted by the work of Symms et al.^59^ The most commonly reported benefits were shorter hospital stay and reduced morbidity.^49^ The main disadvantages described concerned limited exposure, lower success rates compared to the other alternatives, particularly in cases with multiple defects.^25^ Furthermore, Kim et al demonstrated that hearing outcomes were not always improved.^33^

The MCF approach was recommended for the repair of anteromedial defects, particularly those located medial to the ossicular chain or proximal to the petrous apex, as well as large (>1 cm) or multiple defects.^15^, ^49^, ^59^, ^74^ It was also favored in case of recurrence or previous failed repair, tegmen tympani defects, and patients having received previous radiotherapy.^15^, ^49^, ^59^, ^74^ Reported success rates among the different outcomes often exceeded 86%.^49^ The MCF approach provided better visualization and wider exposure of the skull base, facilitating easier instrumentation and application of multilayered dural repairs. This method was associated with lower recurrence rates, likely due to the ability to address multiple defects, while also often preserving or even improving audiologic thresholds.^16^, ^25^, ^49^ The main disadvantage of this approach was a reported increased risk of craniotomy‐related complications, such as dural tears, subdural/epidural hematomas, meningitis, and potential temporal lobe injury, stroke, or seizures.^15^, ^16^, ^25^ Despite the favorable audiologic outcomes reported by some authors, others described complications including sensorineural hearing loss, injury to the vestibular nerve, and facial nerve palsy secondary to geniculate ganglion injury.^25^, ^59^

An iteration of the MCF approach is the combined approach, where to minimize craniotomy size, proponents of this approach advocate for a limited subtemporal craniotomy, centered over the defect identified via mastoidectomy.^25^ The combined approach increased the success rate of the MCF approach and mitigated some of the risks, but required prolonged intraoperative time, while still carrying morbidity associated with a craniotomy.^20^, ^25^, ^56^, ^62^

## Limitations

Although this systematic review provides valuable insights into the surgical approach trends and preferences among surgeons repairing spontaneous MCF CSF leaks, its findings should be interpreted with caution. The overall quality of evidence among the included studies is low, consisting mostly of case series and case‐control studies. Considering the low incidence of recurrence reported, the possibility of reporting bias as well as complication and recurrence underreporting cannot be safely excluded. Only three databases (MEDLINE; via PubMed, EMBASE, and Cochrane Library) were searched, as they are considered to include the majority of published international literature. Articles published in languages other than English were excluded, and non‐English journals were not reviewed, whereas congress abstracts and studies whose full texts were unavailable were not included.

## Conclusion

The MCF approach is the most popular for the repair of lateral skull base sCSF leaks, with the TM and combined approaches having a similar frequency of use. There is no statistically significant difference demonstrating that an approach is superior or safer. Pros and cons of each approach have been discussed, and multiple factors can influence the choice of approach. Further work such as a prospective randomized controlled trial will be useful.

## Author Contributions

**Dimitrios Spinos**, first author, inception and design of project, data review, preparation and review of manuscript; **Panagiotis Varoutis**, contribution to design, data extraction, review of data and analysis, preparation and review of manuscript; **Georgios Geropoulos**, contribution to design, data extraction, review of data and analysis, preparation and review of manuscript; **Georgios Vavoulis**, contribution to design, data extraction, review of data, preparation and review of manuscript; **Georgios Georgountzos**, contribution to design, data extraction, review of data, preparation and review of manuscript; **Nina Rafailia Karela**, contribution to design, data extraction, review of data, preparation and review of manuscript; **Manthia Papageorgakopoulou**, contribution to design, data extraction, review of data, preparation and review of manuscript; **Kyriacos Evangelou**, contribution to design, data extraction, review of data, preparation and review of manuscript; **Jameel Muzaffar**, senior supervision, contribution to data interpretation and analysis, data and manuscript review and preparation; **Wai Sum Cho**, senior supervision, initial inception and design of the project, contribution to data interpretation and analysis, data and manuscript review and preparation.

## Disclosures

### Competing interests

Nil.

### Funding source

Nil.

## Supporting information

Supporting Materials.docx.
