# Supplementary material for: Surgical Outcomes Comparison of Spontaneous Middle Cranial Fossa Cerebrospinal Fluid Leaks: Systematic Review and Meta‐analysis
Source: Otolaryngol Head Neck Surg. 2025 Apr 29;173(1):27–39. doi: 10.1002/ohn.1279 (PMC12207348; doi:10.1002/ohn.1279)

**Supplemental Materials**

Figure 1. Ovid detailed search strategy

Database: Books@Ovid <April 18, 2024>, APA PsycArticles Full Text, Embase <1974 to 2024 April 21>, HMIC Health Management Information Consortium <1979 to April 2024>, Ovid MEDLINE(R) ALL <1946 to April 21, 2024>, CAB Abstracts <1973 to 2024 Week 17>, Journals@Ovid Full Text <April 21, 2024>, APA PsycInfo <1967 to April 2024 Week 4>, APA PsycInfo <1806 to 1966>, Social Policy and Practice <202411>

Search Strategy:

1 middle.mp. [mp=tx, bt, ti, ab, ct, sh, hw, tn, ot, dm, mf, dv, kf, fx, dq, nm, ox, px, rx, an, ui, ds, on, sy, ux, mx, cw, tc, id, tm, pt] (12525896)

2 cranial.mp. [mp=tx, bt, ti, ab, ct, sh, hw, tn, ot, dm, mf, dv, kf, fx, dq, nm, ox, px, rx, an, ui, ds, on, sy, ux, mx, cw, tc, id, tm, pt] (448492)

3 fossa.mp. [mp=tx, bt, ti, ab, ct, sh, hw, tn, ot, dm, mf, dv, kf, fx, dq, nm, ox, px, rx, an, ui, ds, on, sy, ux, mx, cw, tc, id, tm, pt] (180006)

4 lateral.mp. [mp=tx, bt, ti, ab, ct, sh, hw, tn, ot, dm, mf, dv, kf, fx, dq, nm, ox, px, rx, an, ui, ds, on, sy, ux, mx, cw, tc, id, tm, pt] (1699664)

5 skull.mp. [mp=tx, bt, ti, ab, ct, sh, hw, tn, ot, dm, mf, dv, kf, fx, dq, nm, ox, px, rx, an, ui, ds, on, sy, ux, mx, cw, tc, id, tm, pt] (367919)

6 base.mp. [mp=tx, bt, ti, ab, ct, sh, hw, tn, ot, dm, mf, dv, kf, fx, dq, nm, ox, px, rx, an, ui, ds, on, sy, ux, mx, cw, tc, id, tm, pt] (2744925)

7 temporal.mp. [mp=tx, bt, ti, ab, ct, sh, hw, tn, ot, dm, mf, dv, kf, fx, dq, nm, ox, px, rx, an, ui, ds, on, sy, ux, mx, cw, tc, id, tm, pt] (1903278)

8 bone.mp. [mp=tx, bt, ti, ab, ct, sh, hw, tn, ot, dm, mf, dv, kf, fx, dq, nm, ox, px, rx, an, ui, ds, on, sy, ux, mx, cw, tc, id, tm, pt] (3961565)

9 1 and 2 and 3 (24260)

10 4 and 5 and 6 (30467)

11 7 and 8 (135932)

12 9 or 10 or 11 (174159)

13 CSF.mp. [mp=tx, bt, ti, ab, ct, sh, hw, tn, ot, dm, mf, dv, kf, fx, dq, nm, ox, px, rx, an, ui, ds, on, sy, ux, mx, cw, tc, id, tm, pt] (542844)

14 cerebrospinal.mp. [mp=tx, bt, ti, ab, ct, sh, hw, tn, ot, dm, mf, dv, kf, fx, dq, nm, ox, px, rx, an, ui, ds, on, sy, ux, mx, cw, tc, id, tm, pt] (598331)

15 fluid.mp. [mp=tx, bt, ti, ab, ct, sh, hw, tn, ot, dm, mf, dv, kf, fx, dq, nm, ox, px, rx, an, ui, ds, on, sy, ux, mx, cw, tc, id, tm, pt] (2590010)

16 leak.mp. [mp=tx, bt, ti, ab, ct, sh, hw, tn, ot, dm, mf, dv, kf, fx, dq, nm, ox, px, rx, an, ui, ds, on, sy, ux, mx, cw, tc, id, tm, pt] (239871)

17 spontaneous.mp. [mp=tx, bt, ti, ab, ct, sh, hw, tn, ot, dm, mf, dv, kf, fx, dq, nm, ox, px, rx, an, ui, ds, on, sy, ux, mx, cw, tc, id, tm, pt] (1526501)

18 encephalocele.mp. [mp=tx, bt, ti, ab, ct, sh, hw, tn, ot, dm, mf, dv, kf, fx, dq, nm, ox, px, rx, an, ui, ds, on, sy, ux, mx, cw, tc, id, tm, pt] (14578)

19 meningocele.mp. [mp=tx, bt, ti, ab, ct, sh, hw, tn, ot, dm, mf, dv, kf, fx, dq, nm, ox, px, rx, an, ui, ds, on, sy, ux, mx, cw, tc, id, tm, pt] (10475)

20 meningoencephalocele.mp. [mp=tx, bt, ti, ab, ct, sh, hw, tn, ot, dm, mf, dv, kf, fx, dq, nm, ox, px, rx, an, ui, ds, on, sy, ux, mx, cw, tc, id, tm, pt] (2006)

21 otorrhea.mp. [mp=tx, bt, ti, ab, ct, sh, hw, tn, ot, dm, mf, dv, kf, fx, dq, nm, ox, px, rx, an, ui, ds, on, sy, ux, mx, cw, tc, id, tm, pt] (12669)

22 otorrhoea.mp. [mp=tx, bt, ti, ab, ct, sh, hw, tn, ot, dm, mf, dv, kf, fx, dq, nm, ox, px, rx, an, ui, ds, on, sy, ux, mx, cw, tc, id, tm, pt] (1975)

23 14 and 15 and 16 (25756)

24 17 and 23 (5780)

25 13 and 16 (21420)

26 17 and 25 (5390)

27 21 or 22 (14037)

28 17 and 27 (1739)

29 18 or 19 or 20 or 24 or 26 or 28 (30991)

30 12 and 29 (4074)

31 limit 30 to english language [Limit not valid in Books@Ovid,Journals@Ovid,HMIC,Social Policy and Practice; records were retained] (3919)

32 limit 31 to human [Limit not valid in Books@Ovid,Journals@Ovid,HMIC,CAB Abstracts,Social Policy and Practice; records were retained] (3811)

33 remove duplicates from 32 (3287)

34 limit 33 to original articles [Limit not valid in Books@Ovid,Embase,HMIC,Ovid MEDLINE(R),CAB Abstracts,APA PsycInfo,Social Policy and Practice; records were retained] (2202)

Figure 2. Funnel plot analysis for complications in MCF approach


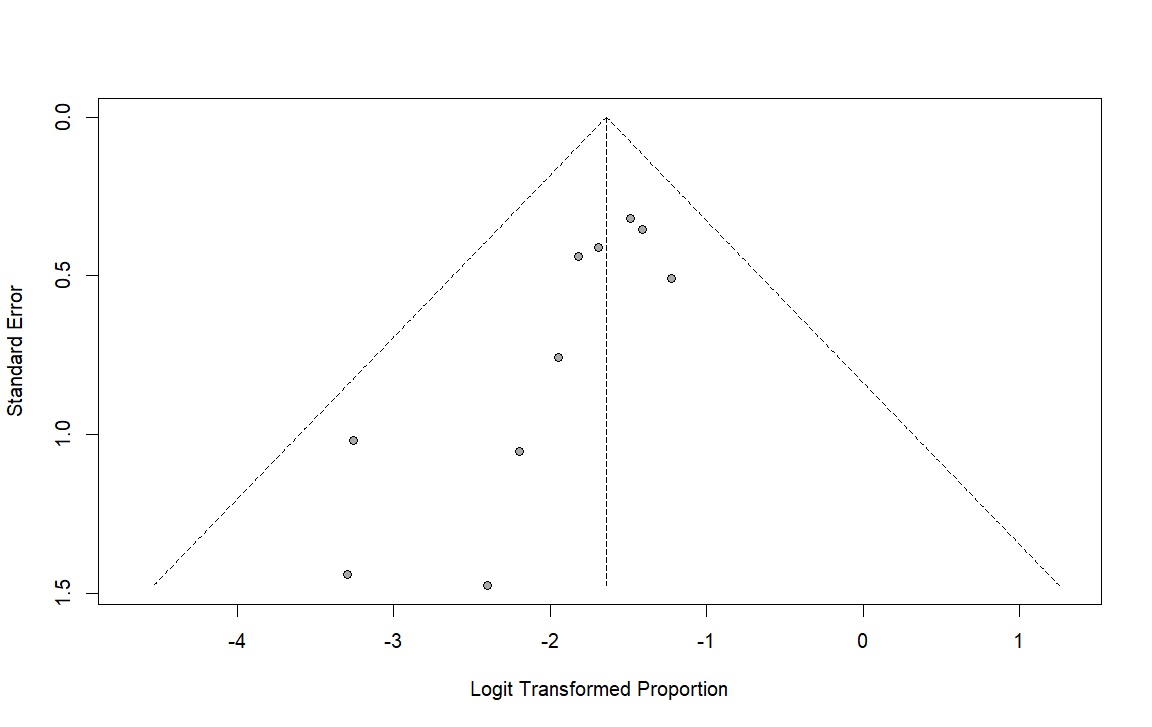


Figure 3. Leave One Out sensitivity analysis for complications in Combined approach


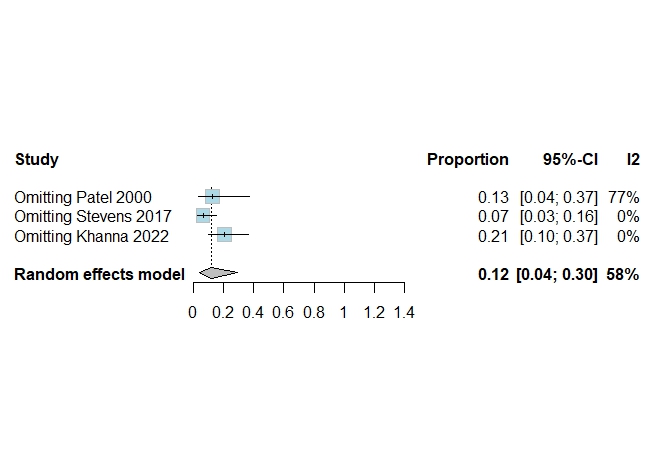


Figure 4. Baujat plot for complications in Combined approach


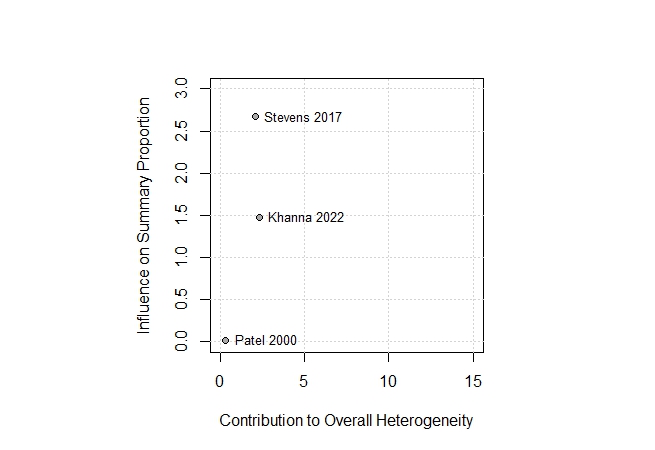


Figure 5. Influential studies for complications in Combined approach


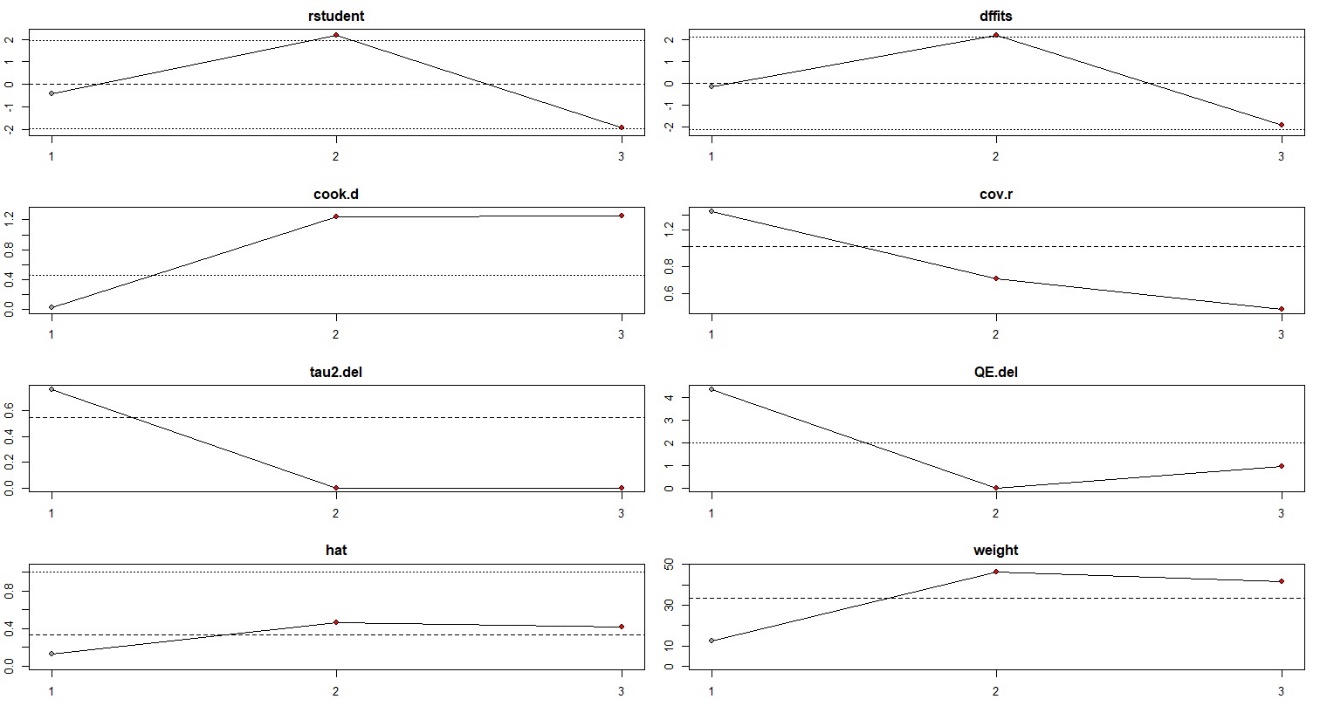


Figure 6. Funnel plot analysis for complications in Combined approach


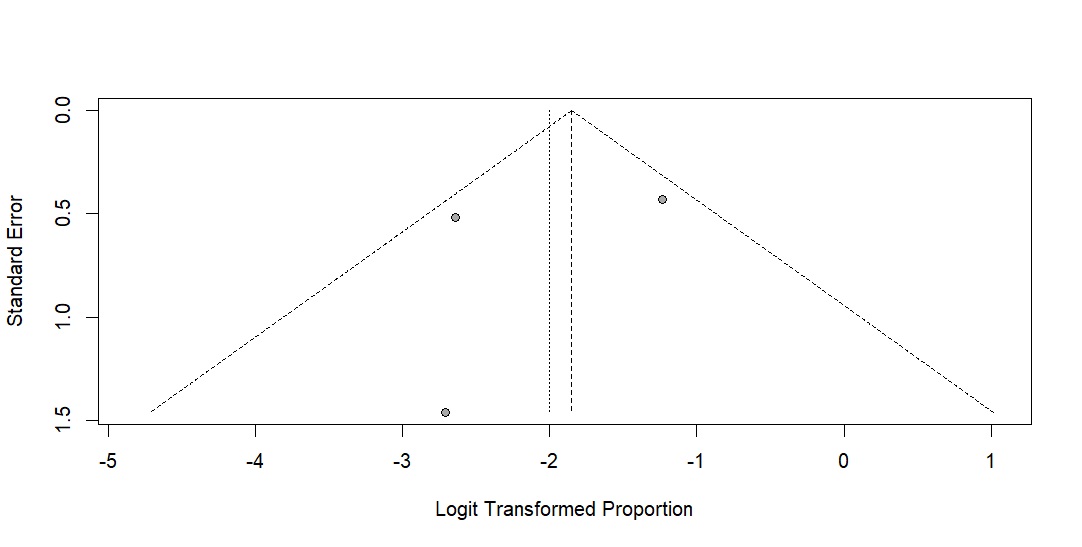


Figure 7. Funnel plot analysis for recurrences in MCF approach


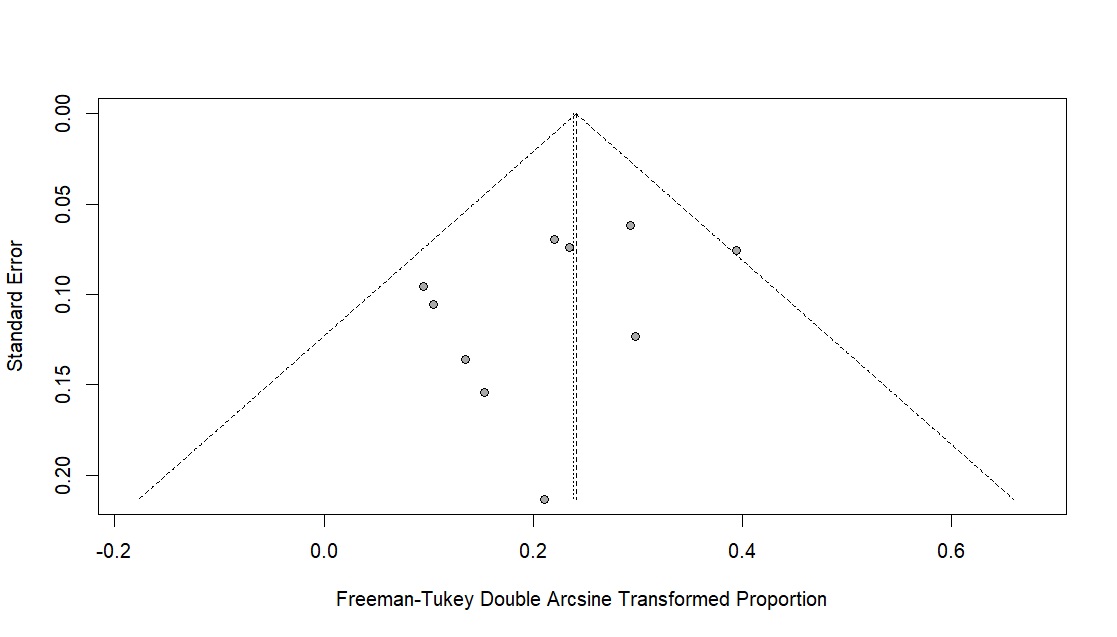


Figure 8. Leave one out analysis for reoperations in MCF approach


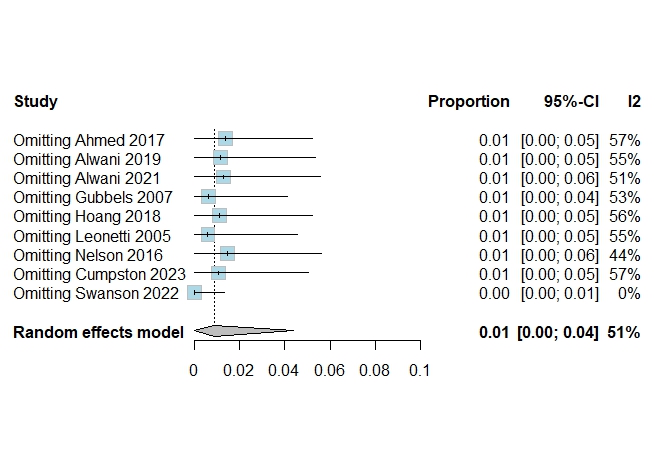


Figure 9. Baujat plot for reoperations in MCF approach


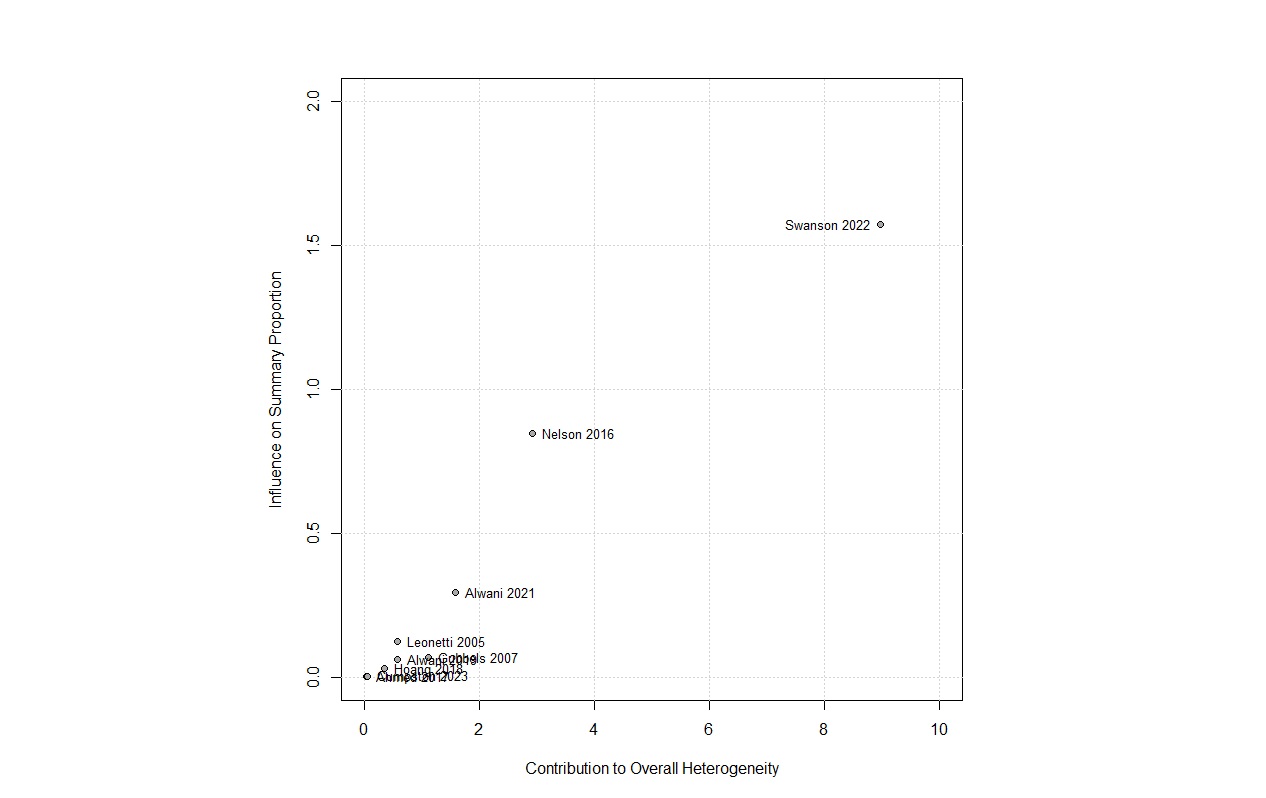


Figure 10. Influential studies for reoperations in MCF approach


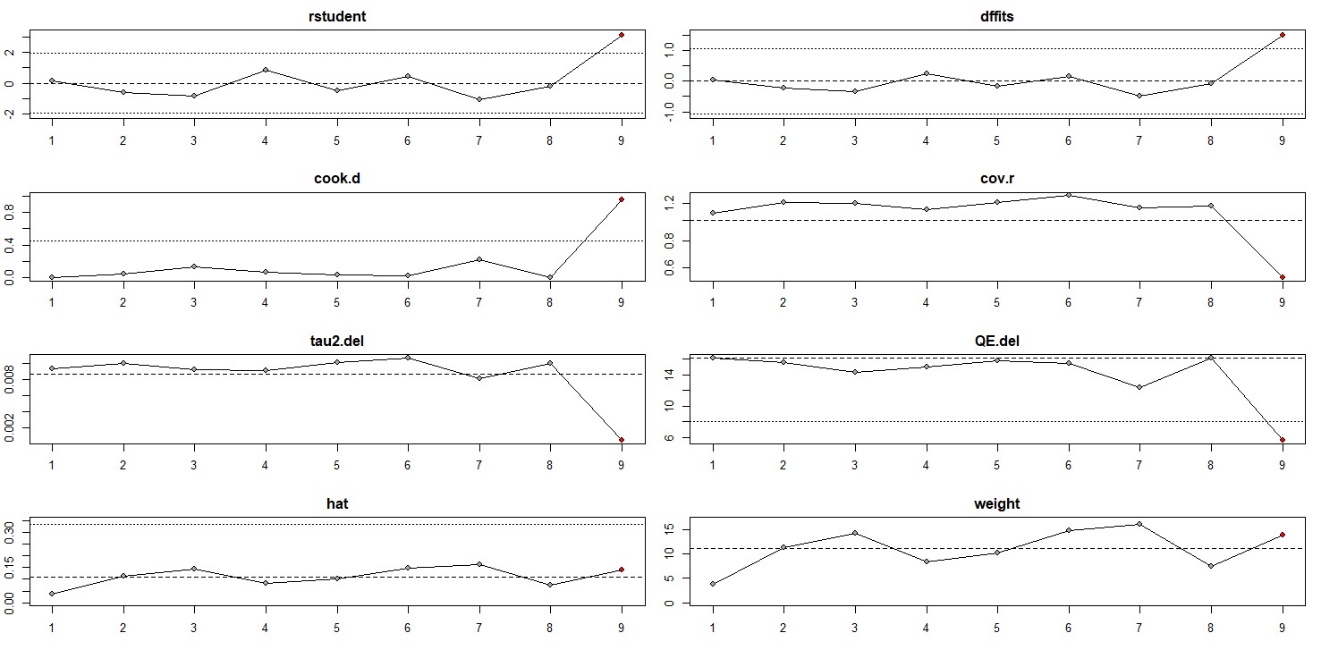


Figure 11. Funnel plot for reoperations in MCF approach


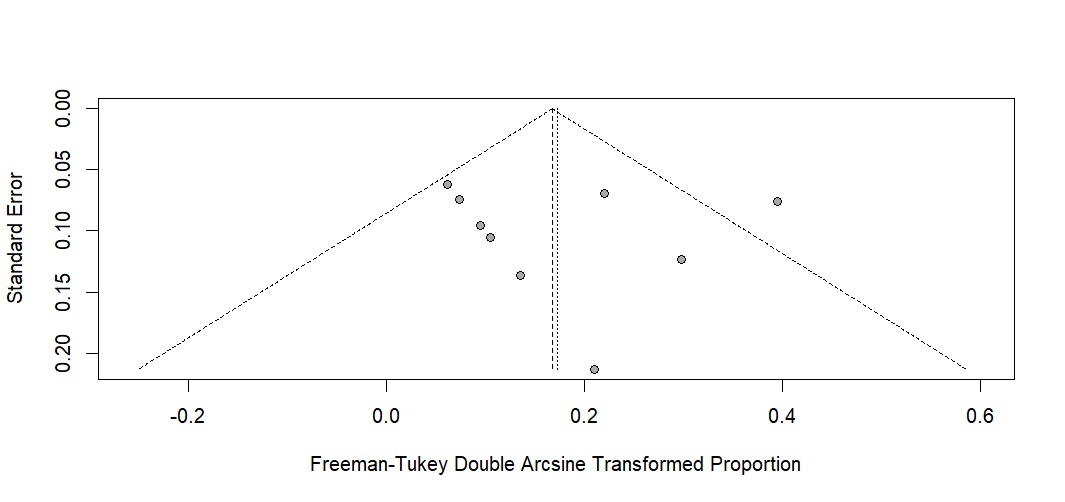

Supplement: Supplementary file 1 — Supporting Materials.docx. [file OHN-173-27-s001.docx]
